# Moderate Static Magnet Fields Suppress Ovarian Cancer Metastasis via ROS-Mediated Oxidative Stress

**DOI:** 10.1155/2021/7103345

**Published:** 2021-12-07

**Authors:** Chao Song, Biao Yu, Junjun Wang, Xinmiao Ji, Lei Zhang, Xiaofei Tian, Xin Yu, Chuanlin Feng, Xinyu Wang, Xin Zhang

**Affiliations:** ^1^High Magnetic Field Laboratory, Hefei Institutes of Physical Science, Chinese Academy of Sciences, Hefei, Anhui 230031, China; ^2^Science Island Branch of Graduate School, University of Science and Technology of China, Hefei, Anhui 230036, China; ^3^Institutes of Physical Science and Information Technology, Anhui University, Hefei, Anhui 230601, China; ^4^International Magnetobiology Frontier Research Center, Science Island, Hefei, Anhui 230036, China

## Abstract

Metastasis is the leading cause of cancer patient death, which is closely correlated with reactive oxygen species (ROS) levels. It is well known that the effects of ROS on tumors are diverse, depending on ROS concentration and cell type. We found that ovarian cancer cells have significantly lower levels of ROS than normal ovarian cells. Moreover, increased ROS levels in ovarian cancer cells can substantially inhibit their migration and invasion ability. Furthermore, the results show that moderate static magnetic field (SMF) can inhibit ovarian cancer cell migration, invasion, and stemness in a ROS-dependent manner. RNA sequencing results confirm that SMFs increased the oxidative stress level and reduced the stemness of ovarian cancer cells. Consistently, the expressions of stemness-related genes were significantly decreased, including hyaluronan receptor (CD44), SRY-box transcription factor 2 (Sox2), and cell myc proto-oncogene protein (C-myc). Furthermore, moderate SMFs provided by a superconducting magnet and permanent magnet have good biosafety and can both inhibit ovarian cancer metastasis in mice. Therefore, our study demonstrates the effects of SMFs on oxidative stress and metastasis in the ovarian cancer cells, which reveals the potential of applying SMF as a physical method in cancer therapy in the future.

## 1. Introduction

Ovarian cancer (OC) is a common gynecologic malignancy and accounts for 5% of female cancer deaths [1]. In 2018, a report showed that there were approximately 22,240 new cases of ovarian cancer diagnosed and 14,070 ovarian cancer deaths in the USA [2]. Due to a lack of specific initial symptoms and limited methods for early diagnosis, most OC patients are diagnosed at advanced stages [3], at which the 5-year survival rate is only ~30% [4]. At advanced OC stages, the leading cause of patient death is metastasis, a complex process regulated by multiple factors. In particular, the presence of cancer stem cells (CSCs) is an essential factor for cancer recurrence, metastasis, and chemotherapy resistance [5–7].

ROS contain a large number of reactive oxygen molecules and oxygen-free radicals, including hydrogen peroxide (H_2_O_2_), hydroxyl radical (OH·), superoxide anion (O_2_^−^·), and hydroxyethyl radical (HER). Although they are essential messengers for multiple signal transduction processes, excessive amount of ROS can cause cellular oxidative stress and cytotoxicity. Moreover, ROS can also inhibit tumor metastasis and reduce cancer stemness. For example, Lu et al. found that high ROS levels induced by *malic enzyme* 2 knockdown could suppress tumor growth, lung metastasis, and peritoneal dissemination in gastric cancer *in vivo* [8]. In addition, elevated ROS levels were intimately related to cell growth and migration reduction in nasopharyngeal carcinoma [9] and non-small-cell lung cancer [10]. Furthermore, Favre et al. confirmed that increased ROS levels could reduce cancer stemness and played an essential role in the transition of quiescent mesenchymal-like states into proliferative epithelial-like states in breast CSCs [11]. Therefore, ROS have been explored as antitumor targets in multiple cancer types [12–14].

Magnetic field (MF) is able to affect ROS levels both *in vitro* and *in vivo* [15]. For example, Van Huizen et al. found that weak SMFs could alter stem cell proliferation and differentiation through regulating ROS levels and the downstream heat shock protein 70 [16]. Recently, two papers both show that SMF or SMF combined with electric field could regulate the redox process and ROS levels, which are essential for their role in alleviating mouse type 2 diabetes [17, 18]. However, the effect of SMF on cancer metastasis is unclear and whether SMF could regulate ROS levels to interfere with cancer metastasis is still unknown.

Here, we investigated the effects of ~0.5 T moderate SMFs, which have been shown to be able to inhibit tumor growth and regulate ROS levels in previous studies [19]. We examined their effects on ovarian cancer cell migration and invasion *in vitro*, as well as ovarian cancer metastasis *in vivo*. We also performed mechanistic studies and found that SMF-induced ovarian cancer metastasis is through elevated ROS levels and oxidative stress, which reduced ovarian cancer cell migration, invasion, and cancer stemness.

## 2. Materials and Methods

### 2.1. Magnetic Field Exposure

In this study, SMFs of ~0.5 T with gradient were provided by superconducting magnet or permanent magnets. A superconducting magnet (Western Superconducting, Xi'an, China) with proper temperature, gas, and humidity control [20] was operated at 9.4 T at the maximum intensity at the center. Our samples were placed in the upper part of the superconducting magnet, where the magnetic field is approximately 0.5 T. Besides, N38 neodymium permanent magnets (NdFeB) were also used to provide SMFs of ~0.5 T (Sans, Nanjing, China). The magnets were placed in a regular cell incubator to treat cells. For mouse exposure, we made a magnetic plate with 12 neodymium magnet cubes (length × width × height = 250 mm × 160 mm × 45 mm). The maximum intensity on the surface of the magnet is also ~0.5 T. To measure the distributions of the magnetic fields at different positions, a magnet analyzer (FE-2100RD, Forever Elegance, China) was used to scan the SMF distribution above the magnets. The sham groups were set up for all experiments. For the superconducting magnet, cells were placed into an identical cellular incubation device without inserting in the magnet. For permanent magnets, the sham groups were set up using unmagnetized neodymium cubes/plates.

### 2.2. Cell Culture

The normal human ovarian cells (IOSE386) and ovarian cancer cells (SKOV3 and HO8910) were obtained from Cell Resource Center of the Shanghai Institutes for Biological Sciences (Shanghai, China). All cells were cultured in RPMI-1640 medium (Corning, USA) containing 10% fetal bovine serum (FBS) (Clark, Germany) and 1% penicillin-streptomycin solution (Hyclone, USA). The cells were maintained in a humidified incubator (Thermo, USA) at 37°C with 5% CO_2_. Same batch of cells was used for the sham and SMF groups. The cell passage numbers were lower than 20 for all experiments.

### 2.3. Analyses of Gene Expression and Transcriptomes

SKOV3 cells were exposed to ~0.5 T SMFs in the superconducting magnet or the sham for 24 hours before their total RNAs were extracted with the RNAeasy™ Animal Long RNA Isolation Kit with Spin Column (R0027, Beyotime). NovoScript Plus All-in-One 1st Strand cDNA Synthesis SuperMix (E047-01A, Novoprotein) was used for RNA reverse transcription, and Novostart SYBR qPCR Supermix Plus (E096-01A, Novoprotein) was used to amplify target gene using specific primers. The thermal cycling conditions comprised an initial denaturation step at 95°C for 30 s, 40 cycles at 95°C for 5 s, and 61°C for 30 s. All the steps were performed according to the manufacturer's instructions. Primers were designed and synthesized by Sangon Biotech Co., Ltd. (Shanghai, China). The detailed primer sequences are shown in Table S1. The expression levels were calculated according to the 2 − ^ΔΔCt^ method [21], where ΔCt is the difference in threshold cycles for the target gene and reference (ACTB), and ΔΔCt is the difference between the ΔCts of the SMF group and sham control. Thus, the expression levels were reported as fold changes relative to the calibrator. The value was used to plot the expression of related genes with the formula 2^−ΔΔCt^.

SKOV3 cells were exposed to ~0.5 T SMFs in the superconducting magnet or the sham for 24 hours before being collected and frozen at -80°C with RNAiso Plus (Takara, Japan). Total RNA was extracted, and a genome-wide transcriptomics analysis was conducted by LC-Bio Technology Co., Ltd (Hangzhou, China). After the final transcriptome was generated, StringTie and Ballgown (http://www.bioconductor.org/packages/release/bioc/html/ballgown.html) were used to estimate the expression levels of all transcripts by calculating FPKM (FPKM = [total_exon_fragments/mapped_reads (millions) × exon_length (kB)]), (command line: ~stringtie-e-B-p 4-G merged.gtf-o samples.gtf samples.bam). The differentially expressed mRNAs were selected with fold change > 1.5 or fold change < 0.65 and *P* value < 0.05 by R package edgeR (https://bioconductor.org/packages/rel ease/bioc/html/edgeR.html) or DESeq2 (http://www.bioconductor.org/packages/release/bioc/html/DESeq2.html). Gene Ontology (GO) and gene set enrichment analysis (GSEA) of the genes with differential expression were performed with LC-Bio Technology Co., Ltd. (https://www.omicstu dio.cn/tool). All gene sets from the MSigDB database gene set were used for the GSEA of the differential genes (http://www.gsea-msigdb.org/gsea/msigdb/index.jsp).

### 2.4. Wound Healing and Transwell Assays

Cell migration was evaluated by the wound healing assay. Cultured cells were seeded in 35 mm culture dishes and grown to full confluence in a complete medium. The cell monolayer was scratched and exposed to ~0.5 T SMFs or 20 *μ*M H_2_O_2_ for 24 or 48 hours. The areas of wound healing were imaged by microscope and quantified by the ImageJ software.

Transwell migration assays were also used to detect the cell migration ability. 5.0 × 10^4^ cells were placed in the upper chamber of 24-well plates with 8.0 *μ*m pore size chamber inserts (Corning, USA) and cultured in serum-free medium with 10 *μ*M NAC or PBS, and 500 *μ*L medium with 10% FBS was added to the lower chamber, which was exposed to ~0.5 T SMFs for 48 hours. At last, cells on the lower surface were fixed with 4% paraformaldehyde, stained with 0.1% crystal violet, and counted under an inverted microscope.

The ability of cell invasion was analyzed by Transwell invasion assay using 24-well plates with 8.0 *μ*m pore size chamber (Corning, USA). The upper surface of the filter was coated with 80 *μ*L of Matrigel (BD Biosciences, San Jose, CA) diluted 1 : 10 in a serum-free medium. The other procedures are the same as the Transwell migration experiment.

### 2.5. Sphere Formation Array

1000 cells were mixed with the stem cell medium containing serum-free DMEM/F12 medium supplemented with 20% B27 (Gibco, USA), 20 ng/mL epidermal growth factor (EGF), and 10 ng/mL basic fibroblast growth factor (bFGF) and seeded in 35 mm ultra-low attachment dishes (Corning, USA). The dishes with seeded cells were exposed to 0.5 T SMF for 12 days to assess their ability of sphere formation.

### 2.6. Intracellular ROS Test

SKOV3 and HO8910 cells (1.0 × 10^5^ cells/mL) were seeded in 35 mm culture dishes and supplied with complete medium. After attachment, the cells were exposed to SMFs for 12 or 24 hours. ROS detection kit (Sigma, USA) containing 2′,7′-dichlorodihydrofluorescein diacetate (DCFH-DA) was used to detect cellular ROS. Cultured cells were incubated with 10 *μ*M DCFH-DA at 37°C for 30 minutes before their ROS levels were evaluated using flow cytometry.

### 2.7. Cell Number

SKOV3 and HO8910 cells (5.0 × 10^4^ cells/mL) were seeded in 35 mm culture dishes and supplied with a complete medium. After attachment, the dishes were placed in 0.5 T SMF for 24 hours, and the cell numbers were counted by flow cytometry. Additionally, cultured cells were seeded in a 96-well plate and supplied with a complete medium with 20 *μ*M H_2_O_2_ for 24, 48, 72, or 96 hours. Cell counting kit-8 (Beyotime, China) was used to assess cell viability.

### 2.8. Animal Experiment

Six-week-old female BALB/c nude mice were obtained from Nanjing Biomedical Research Institute of Nanjing University (Nanjing, China). In this study, all mice were injected with 1.0 × 10^5^ SKOV3 cells intraperitoneally and randomly divided into two groups (sham vs. SMF). The sham group was treated with “fake” magnetic condition, which was used as a control group for magnetic field experiments. In the pilot study, mice were exposed to ~0.5 T SMF in the superconducting magnet (10 hours/day, 7 days/week, 6 weeks) to evaluate the *in vivo* effects of moderate SMF on OC. The food consumption and body weight were recorded during the whole experiment. Serum was collected in a 1.5 mL centrifuge tube and analyzed by an automated biochemical analyzer (HITACHI 7020, Japan). The heart, liver, spleen, lung, kidney, and intestine were collected for imaging by the small animal live imaging system (IVIS Spectrum, PerkinElmer) and further H&E staining and immunohistochemistry analysis. The physiological conditions of mice were monitored by the small animal vital sign monitor (STARR Life Sciences, USA). The signal sensor was placed on the neck of the mice and monitored for 6 minutes. The breath rate, pulse distention, heart rate, and arterial O_2_ were calculated using MouseOx Plus software (STARR Life Sciences, USA). Another set of mice were exposed to the permanent magnet plate for 24 hours/day, 7 days/week, for 6 weeks. Subsequently, all mice were executed to collect tumors in the abdominal cavity. Tumor weight and metastasis nodule numbers were recorded, and tumors were stored at -80°C for further assay. All animal experiments were conducted according to the NIH *Guide for the Care and Use of Laboratory Animals* and carried out strictly in accordance with the related protocols of Anhui Medical University (Hefei, China).

### 2.9. H&E Staining and Immunohistochemistry

All nude mice were dissected to collect organs, including the heart, liver, spleen, lung, and kidney. Then, organs were fixed and processed with formalin to obtain 5 *μ*m thick sections and stained with H&E. Five random areas were examined in each section.

The tumors treated by a permanent magnetic plate were fixed and processed with formalin to obtain 5 *μ*m thick sections. Tissue immunohistochemistry was performed using the antibodies for C-myc (GB13076, Servicebio), Sox2 (GB11249, Servicebio), and CD44 (GB113500, Servicebio). All steps were performed according to the manufacturer's instructions.

### 2.10. Statistical Analysis

All statistical analysis was performed using GraphPad Prism version 8. Data from the experiments were showed as the means ± SEM. The *P* values were calculated using the one-way or two-way analysis of variance (ANOVA) with Bonferroni correction for comparison between three groups or two-tailed unpaired *t*-test for comparison between two groups. *P* < 0.05 was considered statistically significant.

## 3. Results

### 3.1. High Levels of Cellular ROS Inhibit Ovarian Cancer Cell Migration and Invasion

**Figure 1:**
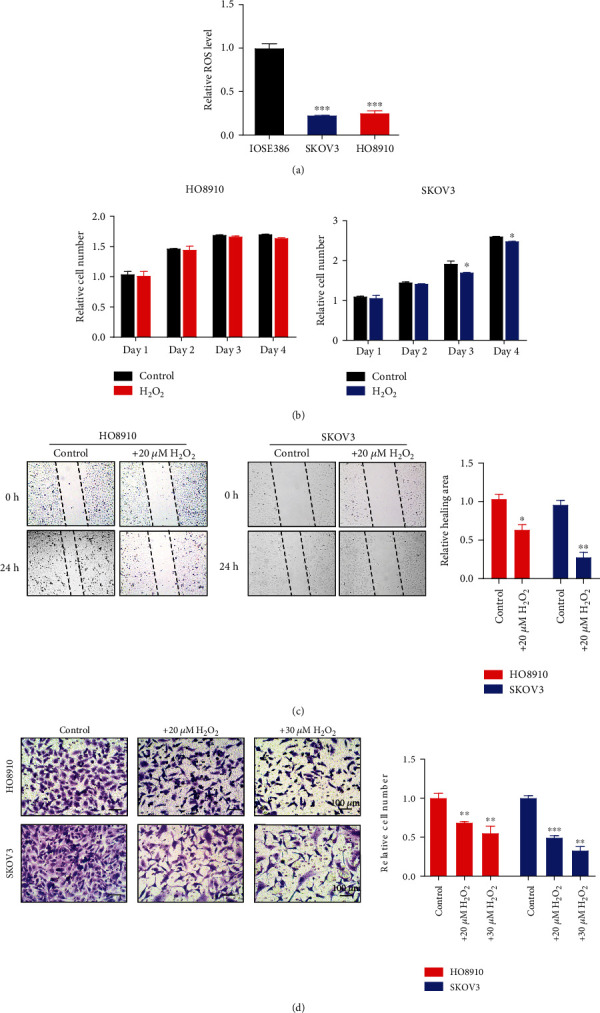
High levels of cellular ROS inhibit ovarian cancer cell migration and invasion. (a) ROS levels of three cell lines (IOSE386, HO8910, and SKOV3) were measured using 10 *μ*M DCFH-DA and flow cytometry. Comparisons were made between the experimental group and the sham control group by Student's *t*-test. (b) The relative cell number of HO8910 and SKOV3 cells treated with or without 20 *μ*M H_2_O_2_. Comparisons were made between the experimental groups and the control groups by two-way analysis of variance (ANOVA) with Bonferroni correction. (c) Wound healing assays of HO8910 and SKOV3 cells treated with or without 20 *μ*M H_2_O_2_. Quantification of the relative healing area is shown on the right. Comparisons were made between the experimental group and the sham control group by Student's *t*-test. (d) Transwell migration assays of HO8910 and SKOV3 cells treated with or without 20 *μ*M H_2_O_2_. Quantification of the relative migrated cells is shown on the right and one-way analysis of variance (ANOVA) with Bonferroni correction for comparison between three groups. ^∗^*P* < 0.05, ^∗∗^*P* < 0.01, and ^∗∗∗^*P* < 0.001.

It is well known that cancer and noncancer cells usually have different levels of ROS. We compared the cellular ROS levels of three ovarian cell lines, including the IOSE386 noncancer cells and HO8910 and SKOV3 cancer cells. Since cell density could also affect cellular ROS levels, we seeded these cells at the same density onto the cell culture plates. Our results show that the ROS levels in IOSE386 noncancer cells are much higher than those in HO8910 and SKOV3 cancer cells (Figure 1(a)). Since excessive ROS are known to cause cell death, which is the key mechanism for some treatment modalities such as radiation therapy, we treated HO8910 and SKOV3 cancer cells with 20 *μ*M H_2_O_2_ for different time points to examine the role of ROS in ovarian cancer cell viability. Our results show that H_2_O_2_ decreased SKOV3 cells in a time-dependent manner but not HO8910 cells (Figure 1(b)). However, it is obvious that the SKOV3 and HO8910 cell migration in the wound healing assays was evidently reduced by H_2_O_2_ (Figure 1(c)). Moreover, the invasive abilities of SKOV3 and HO8910 cells were also inhibited by H_2_O_2_ in a dose-dependent manner (Figure 1(d)). Therefore, the increased H_2_O_2_-induced ROS levels can reduce ovarian cancer cell migration and invasion *in vitro*.

### 3.2. Moderate SMFs Increase Ovarian Cancer Cell ROS Levels and Inhibit Cell Migration

**Figure 2:**
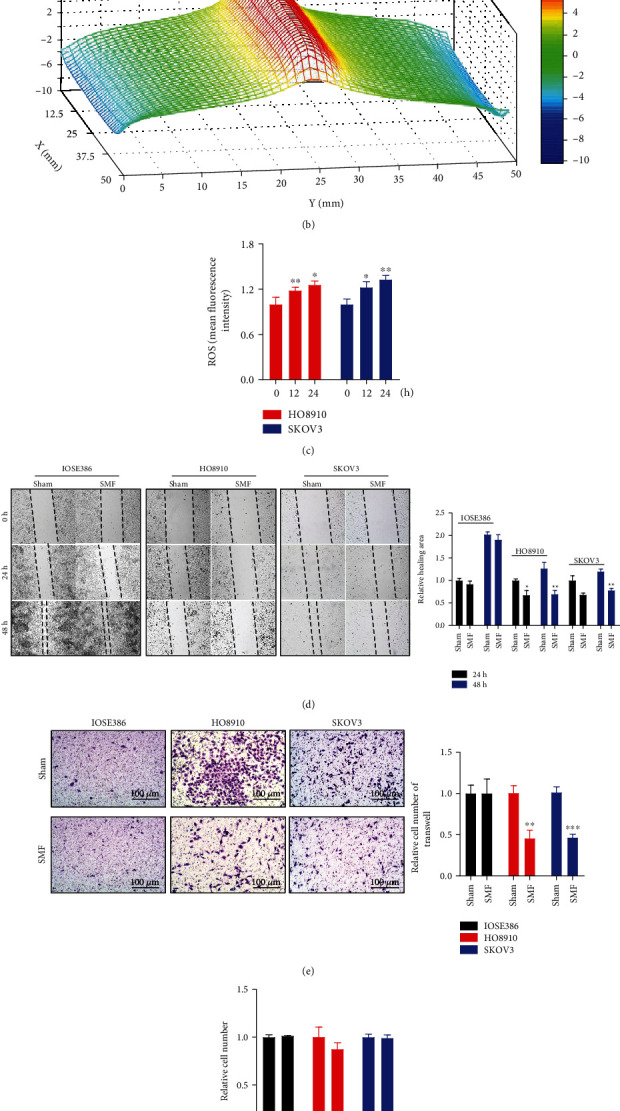
Moderate SMFs increase ovarian cancer cell ROS levels and inhibit cell migration. (a) Illustration of cells exposed to a moderate SMF provided by a permanent magnet. (b) Magnetic field distribution on the magnet surface was measured by a magnet analyzer. The SMF range in the cell culture dish area is 0.1 T~0.5 T. (c) ROS levels of HO8910 and SKOV3 cells exposed to the moderate SMF at different time points and one-way analysis of variance (ANOVA) with Bonferroni correction for comparison between three groups. (d) Wound healing assays of IOSE386, HO8910, and SKOV3 cells exposed to moderate SMF. Quantification of the relative healing area is shown on the right. Comparisons were made between two groups by Student's *t*-test. (e) Transwell invasion assays of HO8910 and SKOV3 cells treated with or without 20 *μ*M H_2_O_2_. Quantification of the invasive cells is shown on the right. Comparisons were made between two groups by Student's *t*-test. (f) Relative cell numbers of IOSE386, HO8910, and SKOV3 cells exposed to moderate SMF for 24 hours. Comparisons were made between the experimental group and the sham control group by Student's *t*-test. ^∗^*P* < 0.05, ^∗∗^*P* < 0.01, and ^∗∗∗^*P* < 0.001.

To examine the effects of moderate-intensity SMF on ovarian cancer cells, we exposed HO8910 and SKOV3 ovarian cancer cells to a moderate-intensity SMF provided by a permanent magnet (Figure 2(a)). The surface of SMF distribution is uneven and ranges from 0.1 T to 0.5 T where the cells were positioned (Figure 2(b)). Our results show that this moderate-intensity SMF could increase cellular ROS levels in HO8910 and SKOV3 cells (Figure 2(c)). After 24 hours of SMF treatment, the cellular ROS levels of HO8910 cells were increased by 23.55% (*P* < 0.05) and SKOV3 cells were increased by 32.88% (*P* < 0.01) (Figure 2(c)). Moreover, wound healing assays show that the migrations of both HO8910 and SKOV3 cells were inhibited by SMFs, but not the IOSE386 noncancerous cells (Figure 2(d)). Similarly, Transwell invasion assays show that the invasions of these two ovarian cancer cell lines were both significantly suppressed by the SMF (54.67% and 53.74% reduction, *P* < 0.01 and *P* < 0.005, respectively), but not the IOSE386 noncancerous cells, which had a low invasion activity (Figure 2(e)). We also examined the effect of this moderate SMF on cell proliferation by examining the cell numbers, which showed no obvious changes after SMF treatment (Figure 2(f)). Taken together, our results show that these moderate SMFs of 0.1 to 0.5 T could increase cellular ROS levels and inhibit ovarian cancer cell migration and invasion *in vitro*.

### 3.3. Moderate SMFs Reduce Ovarian Cancer Stemness

**Figure 3:**
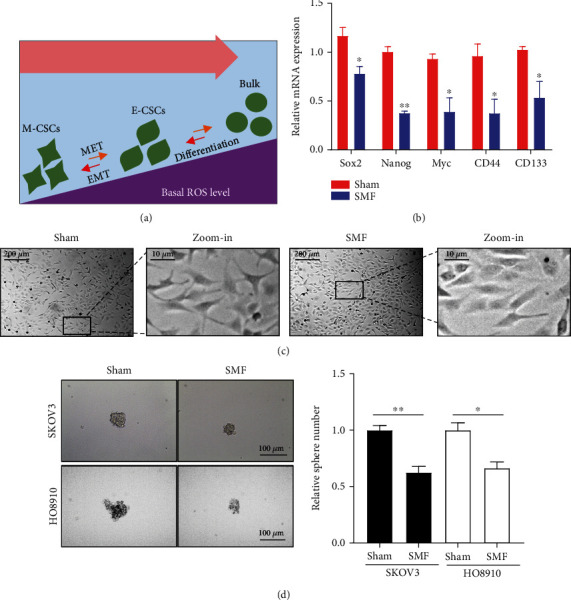
Moderate SMFs reduce ovarian cancer stemness. (a) Illustration of the effects of ROS level on CSCs. (b) The relative mRNA expressions of stemness genes were measured by qPCR, including Sox2, Nanog, C-myc, CD44, and CD133. (c) Representative bright-field images of SKOV3 cells exposed to sham or moderate SMF for 24 h. (d) The sphere number and size were measured in SKOV3 and HO8910 cells treated with SMF for 12 days. All comparisons were made between the experimental group and the sham control group by Student's *t*-test. ^∗^*P* < 0.05 and ^∗∗^*P* < 0.01.

ROS could affect the epithelial-mesenchymal transition (EMT), which promotes the transition of mesenchymal CSCs into epithelial CSCs and then bulk cells [11] (Figure 3(a)). To investigate whether SMFs can reduce cancer stemness, SKOV3 cells were exposed to moderate SMF for 24 hours. Then, the total RNA was extracted and examined by real-time PCR to detect the expression of cancer stem genes (Figure 3(b)). We found that the stemness-related genes were significantly downregulated by SMF treatment, including Sox2, Nanog, C-myc, CD44, and CD133 (Figure 3(b)). For example, the mRNA expression of Nanog, a CSC biomarker, was decreased by 62.48% after SMF exposure (*P* < 0.01) (Figure 3(b)). The mRNA expression of CD44, another CSC marker, was also decreased by 60.94% after SMF exposure (*P* < 0.01) (Figure 3(b)). Moreover, we also observed that the cell morphology of SKOV3 cells changed from mesenchymal-like states to epithelial-like states after SMF exposure (Figure 3(c)). Furthermore, we exposed the HO8910 and SKOV3 cells to SMF for 12 days and detected their sphere-forming ability. The number and size of OC cell spheres were obviously decreased by SMF (Figure 3(d)). These data suggested that ovarian cancer stemness was significantly reduced by this moderate SMF treatment.

### 3.4. Moderate SMFs Inhibit Ovarian Cancer Invasion in a ROS-Dependent Manner

**Figure 4:**
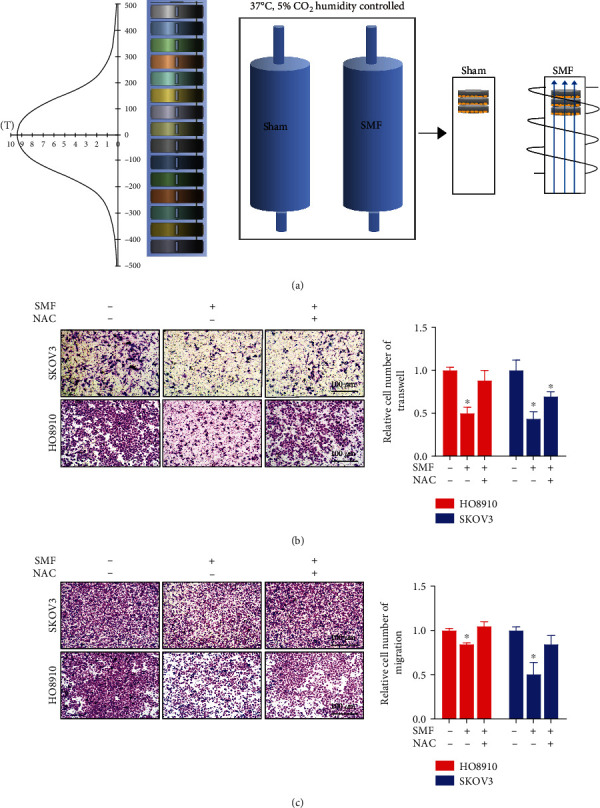
Moderate SMFs inhibit ovarian cancer invasion in a ROS-dependent manner. (a) The SMF intensity distribution inside a superconducting magnet. Cells were placed in the upper part of the superconducting magnet, where the SMF is about 0.5 T. (b) Transwell invasion assays and (c) migration assays of SKOV3 and HO8910 ovarian cancer cells in the absence or presence of SMF and/or NAC. Quantifications of the relative cell numbers on the bottom plates are shown on the right. All comparisons were made between the experimental group and the sham control group by Student's *t*-test. ^∗^*P* < 0.05.

To further examine the effects of moderate SMFs on ovarian cancer cells, we used a superconducting magnet that has a cell culture compatible system (Figure 4(a)) [20]. We placed the cells at the upper part of the magnet where the intensity of SMF is ~0.5 T. Similar to the permanent magnet, this 0.5 T SMF also significantly decreased HO8910 and SKOV3 ovarian cancer cell invasion (Figure 4(b)) and migration (Figure 4(c)) in Transwell assays. To further examine the effects of increased ROS on ovarian cancer metastasis when exposed to moderate SMFs, we used N-acetyl-L-cysteine (NAC), a frequently used reagent to eliminate cellular ROS. Interestingly, the reduction effects of 0.5 T SMF in cell invasion and migration were abolished by NAC (Figures 4(b) and 4(c)), which confirms that SMF reduces ovarian cancer cell invasion and migration via ROS elevation.

### 3.5. RNA-Seq Reveals That Moderate SMFs Regulate Redox Process and Reduce Ovarian Cancer Stemness

**Figure 5:**
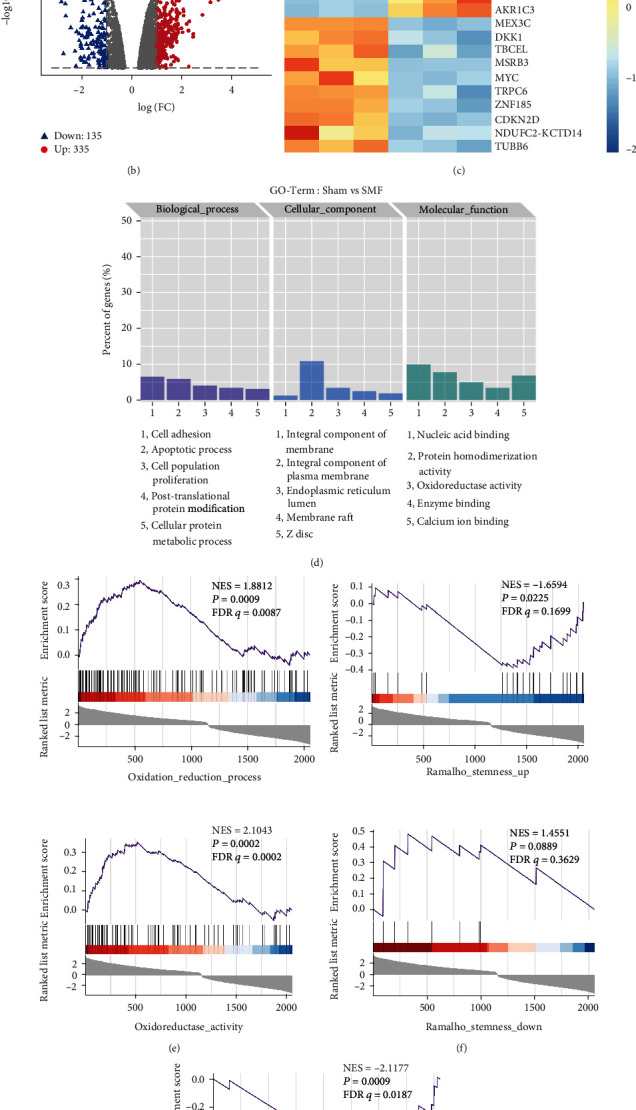
RNA-seq reveals that moderate SMFs regulate the redox process and reduce ovarian cancer stemness. (a) Box plot analysis was used to assess the biological duplication between sample groups. Volcano map (b), heat map of top 20 genes (c), and GO term (d) of the differential expression genes by RNA seq. (e–g) The GSEA was used to assess the relation of the differential genes and gene sets from the MSigDB database gene set, including OXIDATION_REDUCTION_PROCESS, OXIDOREDUCTASE ACTIVITY, PECE_MAMMARY_STEM_CELL_UP, PECE_MAMMARY_STEM_CELL_DOWN, RAMALHO_STEMNESS_UP, and RAMALHO_STEMNESS_DOWN.

To assess the effects of SMF on the ovarian cancer transcriptomes, we placed the SKOV3 ovarian cancer cells in the upper part of the superconducting magnet, where the SMF is approximately 0.5 T, or in the sham device. Six dishes of cells were treated for 24 hours before their total RNA was extracted for RNA sequencing analysis. The box plot analysis showed a good biological duplication among the samples (Figure 5(a)). Then, we analyzed and selected 467 genes with significant differential expressions, including 332 upregulated and 135 downregulated genes (Figure 5(b)). Moreover, the gene heat map indicated that the nuclear factor E2-related factor 2- (NRF2-) mediated antioxidant genes were activated significantly. For example, glutathione peroxidase 1 (GPX1) and aldo-keto reductase family 1 members (AKR1B1, AKR1C1, AKR1C2, and AKR1C3) are all in the top 20 of the most upregulated expressions (Figure 5(c)). GO term suggested that the differentially expressed genes are closely associated with oxidoreductase activity, protein homodimerization activity, cell adhesion, apoptotic process, cell population proliferation and cell membrane, etc. (Figure 5(d)). Furthermore, we found that the genes were enriched in the OXIDATION_REDUCTION_PROCESS (NES = 1.8812, *P* = 0.0009) and OXIDOREDUCTASE ACTIVITY (NES = 2.1043, *P* = 0.0002) (Figure 5(e)) from GSEA, indicating that moderate SMF participates in the redox process regulation.

Interestingly, the GSEA results also indicated that the differential genes were enriched in the OUELLET_OVARIAN CANCER_INVASIVE_VS LMP_UP (NES = −2.14, *P* = 0.0013), but not the OUELLET_OVARIAN CANCER_INVASIVE_VS LMP_DOWN (NES = 0.87, *P* = 0.6665) (Supplementary Figure 1), indicating that SMFs could be closely related to the ovarian cancer metastasis. Since CSCs have unique abilities, including high metastasis and chemotherapy drug resistance, the reduction of cancer stemness will be an effective antitumor strategy. To examine the effects of moderate SMFs on CSCs in ovarian cancer, we analyzed the correlation between the differential genes and cancer stemness by the GSEA. It is interesting that the genes were enriched in the PECE_MAMMARY_STEM_CELL_UP (NES = −2.1177, *P* = 0.0009) and RAMALHO_STEMNESS_UP (NES = −1.6594, *P* = 0.0225). Consistent with the results at cellular level, the GSEA also showed that SMF-induced gene expressions were negatively related to the upregulated genes of stem cells, which suggested moderate SMFs could reduce cancer stemness in ovarian cancer (Figures 5(f) and 5(g)).

### 3.6. Moderate SMFs Inhibit Ovarian Cancer Metastasis in Mice

**Figure 6:**
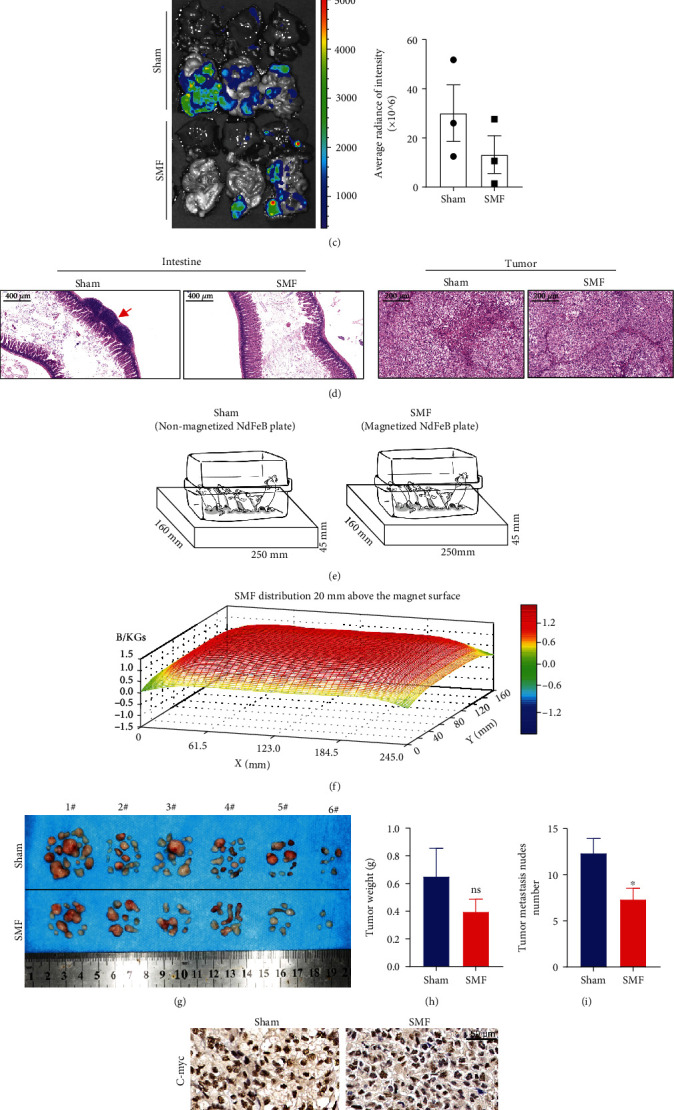
Moderate SMFs inhibit ovarian cancer metastasis in mice. Mice bearing ovarian cancer were exposed to moderate SMFs using a superconducting magnet (a–d) or a permanent magnet plate (e–j). (a) Mice bearing ovarian cancer were exposed to the sham or SMF conditions using a superconducting magnet. The mice were exposed for 10 hours/day and 7 days/week for 6 weeks. (b) Mice were examined for metastasis at the end of the experiment. (c) Heart, liver, spleen, lung, kidney, and intestine in mice were imaged by IVIS spectrum. (d) Representative HE staining images of the tumor and intestinal tissues sections. (e) The mice bearing OC were exposed to an unmagnetized sham NdFeB or a magnetized NdFeB plate for continuous 6 weeks. (f) The magnetic field distribution was scanned by a magnet analyzer at 20 mm above the magnetic surface. (g–i) All tumor nodules from the abdominal cavity of mice were collected at the end of the experiment to imaged (g), weighted (h), and counted (i). (j) The immunohistochemistry of tumor tissue sections was used to detect the expressions of C-myc, Sox2, and CD44. All comparisons were made between the experimental group and the sham control group by Student's *t*-test. ^∗^*P* < 0.05; ns: not significant.

To evaluate the effect of SMF on ovarian cancer metastasis *in vivo*, we injected 1.0 × 10^5^ SKOV3 cells into the abdominal cavity of 24 nude mice and exposed them to moderate-intensity SMFs by a superconducting magnet (Figures 6(a)–6(d)) or by a NdFeB permanent magnet plate (Figures 6(e)–6(j)). Both SMFs are upward direction and within the range of 0.1 ~ 0.5 T.

For the superconducting magnet group (Figures 6(a)–6(d)), the mice were randomly divided into two groups and exposed to the sham condition or SMF for 10 hours/day, 7 days/week for 6 weeks (total of 420 hours). We recorded food and water consumption as well as mouse body weight every week and found no significant changes after SMF exposure (Supplementary Figures 2(a)–2(c)). We also monitored their vital signs at the last pre- and post-exposure. It seems that SMF can increase arterial O_2_ while keeping the breath rate stable, and no other significant differences were found (Supplementary Figure 2(d)). In the 6^th^ week, all mice were executed to collect serum and organs. No significant differences were observed in the blood chemistry analysis, including triglyceride (TG), lactate dehydrogenase (LDH), total protein (TP), alanine aminotransferase (ALT), aspartate aminotransferase (AST), creatine kinase (CK), uric acid (UA), and creatinine (CREA) (Supplementary Figure 2(e)). We also performed H&E staining for the heart, liver, spleen, lung, and kidney. Similarly, no obvious changes were found in comparison with the sham groups (Supplementary Figure 2(f)). These results demonstrate the biosafety of moderate SMF exposure for 420 hours on ovarian metastatic cancer-bearing mice. For the metastatic cancer nodules, a number of tumor nodules were found in the abdominal cavity of sham group mice, especially in the intestine and liver tissues (Figure 6(b)). In contrast, moderate SMFs significantly reduced the tumor growth and metastasis in mice (Figure 6(c)). Moreover, the histological analysis confirmed to the reduction of metastasis nodules in the intestine (Figure 6(d)).

For the permanent magnet group (Figures 6(e)–6(j)), mice were randomly divided into two groups and exposed to the unmagnetized sham control plate or magnetized SMF plate for 24 hours/day, 7 days/week for 6 weeks (total of 1008 hours) (Figure 6(e)). The magnetic field distribution of the magnetized plate was scanned by a magnetic analyzer at 2 mm above the magnet plate, where the mouse bodies were located (Figure 6(f)). We collected total tumor nodules in mice (Figure 6(g)) and found that although moderate SMFs did not obviously affect the weight of these tumor metastasis nodules, the tumor nodule number was significantly reduced (Figures 6(h) and 6(i)). To examine the effect of SMF on cancer stemness in mice, we used immunohistochemistry staining to detect the expressions of C-myc, Sox2, and CD44. Consistent with the *in vitro* cell assay results, the expressions of stemness genes were obviously downregulated by SMF treatment, confirming that SMF could reduce cancer stemness and metastasis in mice.

## 4. Discussion and Conclusion

**Figure 7:**
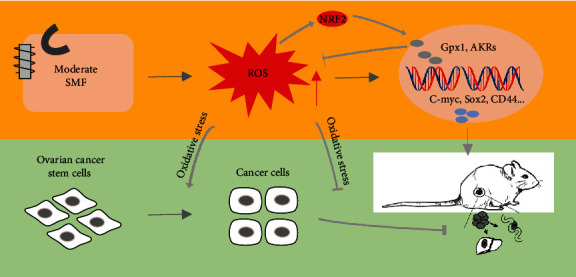
The mechanical diagram of SMF suppressing ovarian cancer metastasis. SMFs suppress the OC metastasis *in vivo* and *in vitro* through regulating ROS level and oxidative stress, which inhibits the transition of normal cancer cells to CSCs.

Our results show that the moderate SMFs we used here could increase the ROS levels of ovarian cancer cells to regulate the expression of antioxidant and stemness genes, which inhibits CSC transition into bulk cells (Figure 7). Consequently, the ovarian cancer metastasis in mice is significantly inhibited by these moderate SMFs.

### 4.1. ROS Levels and Cancer Metastasis

We compared the ROS levels in IOSE386 normal ovarian epithelial cells and SKOV3 and HO8910 ovarian cancer cells and found that the ROS levels in ovarian cancer cells are much lower than those of normal ovarian cells. Low ROS have been indicated to be critical for the self-renewal of stem cells [22, 23]. Diehn et al. showed that normal mammary epithelial stem cells have lower ROS levels than their mature progeny cells. Moreover, subsets of CSCs in some breast tumors contained lower ROS levels than the nontumorigenic cells [24]. In the meantime, increased ROS promoted the transition of CSCs into the normal cancer cells and inhibited tumor metastasis in breast cancer and ovarian cancer [11, 25]. Clearly, altering ROS levels could affect the states of CSCs. Our results show that moderate SMFs can increase ROS levels in ovarian cancer cells and reduce their stemness, which inhibits cancer metastasis.

However, it is well known that ROS are involved in cell growth, differentiation, progression, and death; the effects of altering ROS levels in cancer cells are complicated. ROS-mediated oxidative stress can cause cell death, which is the cytotoxicity mechanism caused by many treatment modalities, including radiotherapy and some chemotherapy drugs. Cancer and noncancer cells often have different levels of ROS, but the relationship between ROS levels and cancer cells is complicated. In many cases, the cancer cells have higher ROS levels than noncancer cells. However, we found that for ovarian cells, the ovarian cancer cells have ~10 times lower ROS levels than normal ovarian cells. The increased ROS levels by either H_2_O_2_ or moderate SMF treatment can reduce their stemness and metastasis.

### 4.2. The Differential Effects of SMFs on ROS Levels

Magnetic field can control the movement and transfer of electrons, so as to manipulate the unpaired electrons in free radicals, which provides a theoretical basis for cellular ROS regulation by SMF [26, 27]. It has been shown that SMFs can affect cellular ROS levels, but the results are variable in different studies [15]. Many studies show that SMFs can increase cellular ROS levels. For example, Nicola et al. found that 6 mT SMF for 2 hours triggered the increase of ROS in human monocyte tumor cells U937 [28]. Also, Calabrò et al. and Vergallo et al. confirmed that the cellular ROS, H_2_O_2_, and ·O_2_^−^ could be elevated by 6 mT~232 mT SMFs in human neuroblastoma SH-SY5Y cells [29, 30]. In addition, 1.2 T high-gradient magnetic field treatment for 24 hours significantly increased cellular ROS in human monocytic leukemia cells THP-1 [31]. It is similar that ROS levels are increased by 8.5 T SMFs in human-hamster hybrid A(L) cells, mitochondria-deficient rho(0) A(L) cells, and double-strand break repair-deficient cells XRS-5, as well as mouse embryonic stem (ES) cells and derived cells [32]. Meanwhile, our previous study showed that the ROS levels were obviously increased by 9.4 T SMF, causing cell cycle arrest in human lung cancer cells A549 [20]. However, multiple studies demonstrate SMFs could reduce cellular ROS levels. For example, Van Huizen et al. found that weak SMFs (<1 mT) altered stem cell proliferation and differentiation through decreasing ROS levels [16]. Moreover, Carter et al. showed that 3 mT SMF in the combination with a static electric field could treat type 2 diabetes (T2D) through regulating the redox process and reducing cellular ROS [17]. Additionally, the reductions of ROS levels were found in human peripheral blood neutrophils [33] and bronchial epithelial cells [34] when exposed to 60 mT and 389 mT SMFs, respectively. In our previous study, exposure to SMFs of 0.1 T~0.5 T could reduce ROS levels and improve gut microbes, which improved T2D mice [18].

Various factors could result in these differential effects of SMF on cellular ROS levels, including SMF intensity, gradient, exposure time, tissue type, or cell type. For instance, human breast cancer cell lines (MCF-7 and MDA-MB-231) were exposed to 1 T SMF for one day, and the results showed a reduction of ROS levels, while the exposure to 10 mT SMF for one day increased ROS levels in MCF-7 [35, 36]. Moreover, Csillag et al. placed human lung cancer cells (A549) in 389 mT SMF for 30 minutes and observed an obvious decrease in ROS levels [34]. On the contrary, our previous study showed that 9.4 T SMF could significantly increase A549 cellular ROS levels [20]. It is obvious that SMFs with different parameters could generate various effects on cancer cells. It seems that lower intensities and gradient SMFs often tend to decrease cellular ROS levels, while higher intensities and gradient SMFs tend to increase cellular ROS levels, but it is not always the case. SMF exposure time, cell type, the potential heat effect of some electromagnetic devices, and specific types of ROS (H_2_O_2_, OH·, or O_2_^−^·, etc.) are also important factors that contribute to the differential effects of SMF on cellular ROS levels, which unquestionably still needs further systematic investigations.

### 4.3. SMF and Cancer

SMF is a safe physical method, even for strong SMFs that are ~10 times higher than the moderate SMFs we used in this study [37–40]. Here, we have also shown that exposure of ovarian cancer-bearing mice to 0.1 T~0.5 T moderate SMFs for 420 hours does not have any safety issues. Moreover, it has been demonstrated by multiple studies that SMFs have some tumor growth inhibition effects [20, 41, 42]. The mechanism involves microtubule assemble disturbance, epidermal growth factor receptor (EGFR) membrane protein orientation and activation, DNA synthesis inhibition, etc. [43–45].

In the current study, although the ovarian cancer cell growth was not obviously affected, we found that moderate SMF could increase ROS levels and oxidative stress in ovarian cancer cells to suppress their stemness, which inhibits cancer metastasis. The effects of MFs on cancer metastasis have always been a hot spot of people's attention. In 2002, Tofani et al. found that 5.5 mT-modulated MF (static with a superimposition of extremely low-frequency fields at 50 Hz) significantly inhibited tumor growth and metastasis in breast cancer MDA-MB-435 [46]. In 2007, Sommer et al. exposed AKR/J mice bearing lymphoma to an electromagnetic field and showed an obvious reduction in tumor metastasis [47]. Moreover, a report in 2009 showed that 42 mT SMF with a MF (150~300 nT, 1~16.5 Hz) obviously suppressed metastasis in Ehrlich ascites cancer [48]. In addition, Nie et al. found that a rotating magnetic field of ~0.4 T and 7.5 Hz could inhibit metastasis in melanoma B16-F10 cells in 2013 [49]. However, the study of alone SMF affecting cancer metastasis is insufficient. There was only one study reported that a hypomagnetic field could reduce the migration and invasion of human neuroblastoma SH-SY5Y [50].

In summary, our work reveals that moderate SMFs could suppress ovarian cancer metastasis *in vivo* and *in vitro*. Cellular studies show that SMF inhibits cell migration and invasion and reduces cancer stemness in ovarian cancer cells. Animal studies show that moderate SMFs could suppress ovarian cancer metastasis *in vivo*. Mechanistically, we show that SMF increases cellular ROS levels and oxidative stress, which promotes the transition of CSCs into normal cancer cells. Therefore, our study demonstrates the potential to develop SMF as a physical tool for cancer therapy in the future.

## Data Availability

The data used to support the findings of this study are included within the article.

## Supplementary Materials

Supplementary Materials Supplementary Figure 1: the GSEA of the differential genes. Supplementary Figure 2: biosafety evaluation of mice exposed to the superconducting magnet. Table S1: sequence-based reagents for analyses of gene expression.
