# Supplementary material for: Moderate Static Magnet Fields Suppress Ovarian Cancer Metastasis via ROS-Mediated Oxidative Stress
Source: Oxid Med Cell Longev. 2021 Dec 7;2021:7103345. doi: 10.1155/2021/7103345 (PMC8670934; doi:10.1155/2021/7103345)
Supplement: Supplementary Materials — Supplementary Figure 1: the GSEA of the differential genes. Supplementary Figure 2: biosafety evaluation of mice exposed to the superconducting magnet. Table S1: sequence-based reagents for analyses of gene expression. [file 7103345.f1.docx]

**Supplementary Materials**


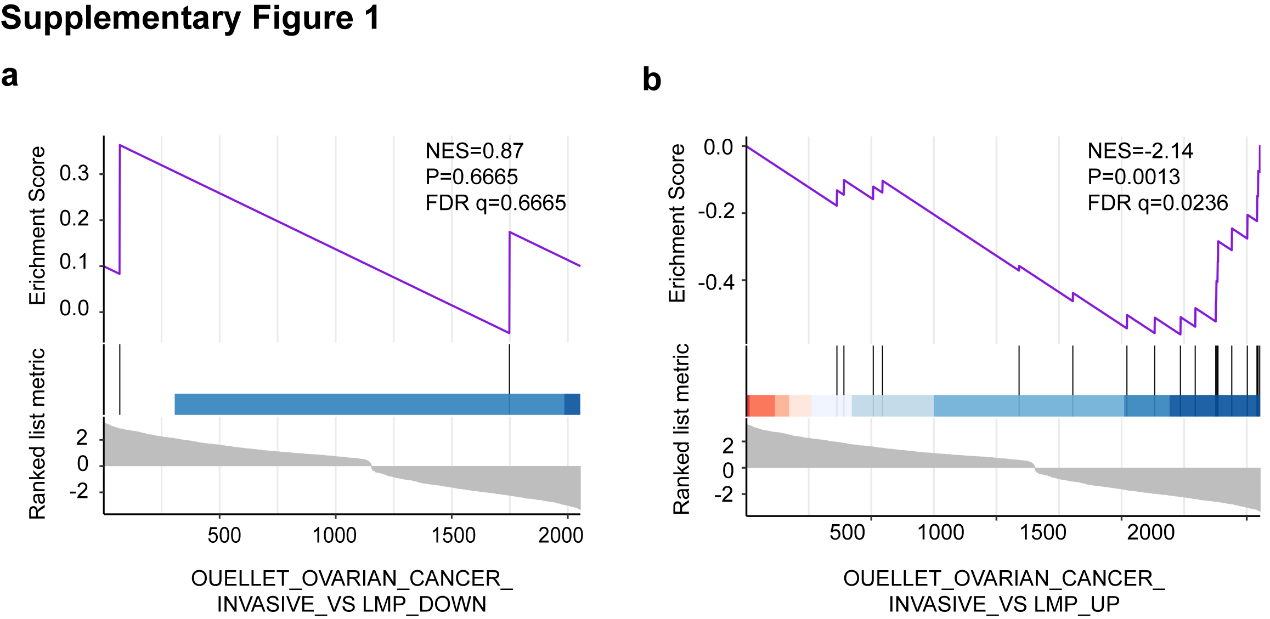


**Supplementary Figure 1: The GSEA of the differential genes.** The GSEA was used to assess the relation of the differential genes and the gene sets including OUELLET_OVARIAN CANCER_INVASIVE_VS LMP_UP (a) and OUELLET_OV ARI IAN CANCER_INVASIVE_VS LMP_DOWN (b).


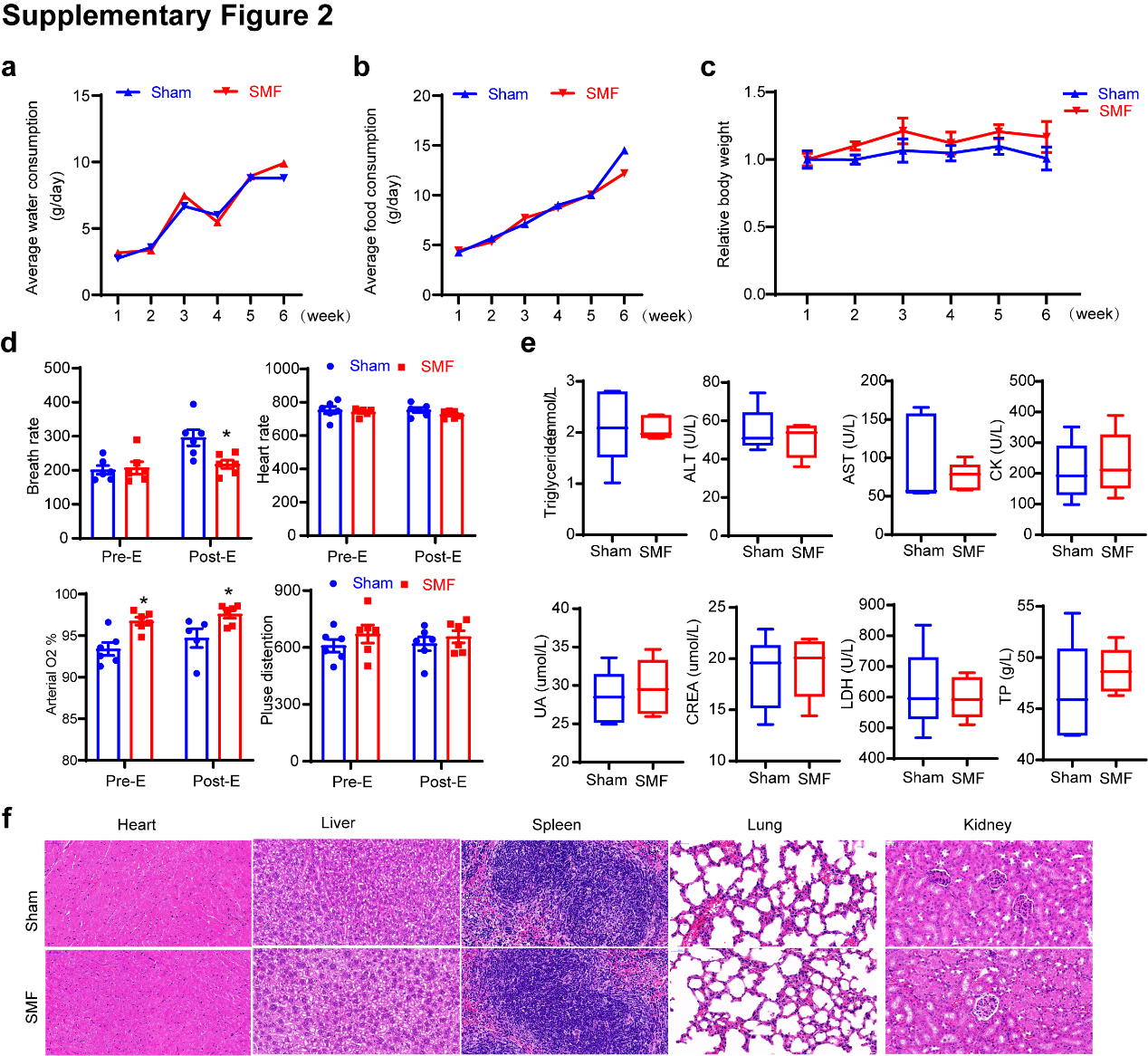


**Supplementary Figure 2: Biosafety evaluation of mice exposed to the superconducting magnet.** The average water (a) and food consumption (b), and body weight (c) were recorded every week, the *P*-values were calculated using the one-way analysis of variance (ANOVA) with Bonferroni correction for comparison between three groups. The heart rate, breath rate, pulse distention and arterial O_2_ (d) were monitored at the last pre- and post-SMF exposure (Pre-E and Post-E). Blood biochemical analysis (e) was performed for triglyceride, ALT, AST, CK, UA, CREA, LDH and TP. And main organs were collected to preform H&E staining for heart, liver, spleen, lung and kidney (f). All comparisons were made between two groups using a Student’s *t*-test. **P* < 0 05, ***P* < 0 01.

Table S1 Sequence based reagents

| Name | Sequence | Supplier |
| --- | --- | --- |
| H-Nanog | F: GATGCCTCACACGGAGACT  R: TTTGCGACACTCTTCTCTGC | Sangon, China |
| H-Sox2 | F: TGCTGCCTCTTTAAGACTAGGAC  R: CCTGGGGCTCAAACTTCTCT | Sangon, China |
| H-CD44 | F: ACAAGCACAATCCAGGCAACTCC  R: TGGTGTTGTCCTTCCTTGCATTGG | Sangon, China |
| H-C-myc | F: CGACGAGACCTTCATCAAAAAC  R: CTTCTCTGAGACGAGCTTGG | Sangon, China |
| H-CD133 | F: GTGGCGTGTGCGGCTATGAC  R: CCAACTCCAACCATGAGGAAGACG | Sangon, China |
| H-β-Actin | F: CTGTCCCTGTATGCCTCTG  R: ATGTCACGCACGATTTCC | Sangon, China |
